# Form-specific selenium supplementation in poultry: linking bioefficacy to mechanisms and precision applications

**DOI:** 10.1186/s40104-026-01436-5

**Published:** 2026-06-13

**Authors:** Jianmin Zhou, Uchechukwu Edna Obianwuna, Yu Fu, Vivian U. Oleforuh-Okoleh, Jesse Oluwaseun Ayantoye, Mohamed Shafey Elsharkawy, Kai Qiu, Haijun Zhang, Guanghai Qi, Shugeng Wu

**Affiliations:** 1 Key Laboratory of Feed Biotechnology, Ministry of Agriculture and Rural Affairs, Institute of Feed Research, Chinese Academy of Agricultural Sciences, Beijing, 100081 P. R. China; 2 Department of Animal Science, Faculty of Agriculture, Rivers State University, P.M.B. 5080, Nkpolu-Oroworukwo, Port-Harcourt, Nigeria; 3 Institute of Animal Sciences, Chinese Academy of Agricultural Sciences, Beijing, 100193 China; 4 Animal Production Department, National Research Centre, Dokki, Giza, Egypt

**Keywords:** Biofortification, Oxidative stress, Poultry nutrition, Precision nutrition, Selenium, Selenomethionine

## Abstract

Selenium (Se) supplementation in poultry is shifting from deficiency prevention to precision use aimed at measurable improvements in resilience, product quality, and biofortification. Evidence increasingly indicates that, at comparable total dietary Se, bioefficacy varies primarily with chemical form, exposure definition, and retention kinetics. Inorganic salts can correct deficiency and support basal selenoprotein activity, but they provide limited reserve formation. By contrast, selenomethionine (SeMet)-type inputs, including SeMet and OH-SeMet, can build protein-bound Se pools that stabilize Se supply during prolonged stress and are associated with measurable outcomes such as improved feed conversion under heat stress and more predictable egg or tissue enrichment. Biotransformation-derived and engineered systems may provide additional advantages in specific contexts, particularly for mucosal delivery or barrier-related outcomes, but their effects remain strongly product- and formulation-dependent. Mechanistically, source-specific effects reflect differences in Se delivery to selenoprotein synthesis and in the stability of redox and inflammatory control under challenge. A central implication is that matched nominal Se doses do not necessarily represent biologically equivalent exposure, and maximal Se deposition does not necessarily coincide with maximal functional benefit. Thus, deposition targets should be distinguished from functional outcomes such as performance, product quality, and physiological resilience. This review integrates form-specific bioefficacy with mechanistic and application-level evidence and proposes a goal-based precision framework to align Se source and dose with defined production or physiological objectives. Future progress will depend on rigorous source characterization, clearer endpoint alignment, and layered monitoring of exposure, status, and function to enable more predictive and actionable Se use in poultry systems.

**Graphical Abstract:**

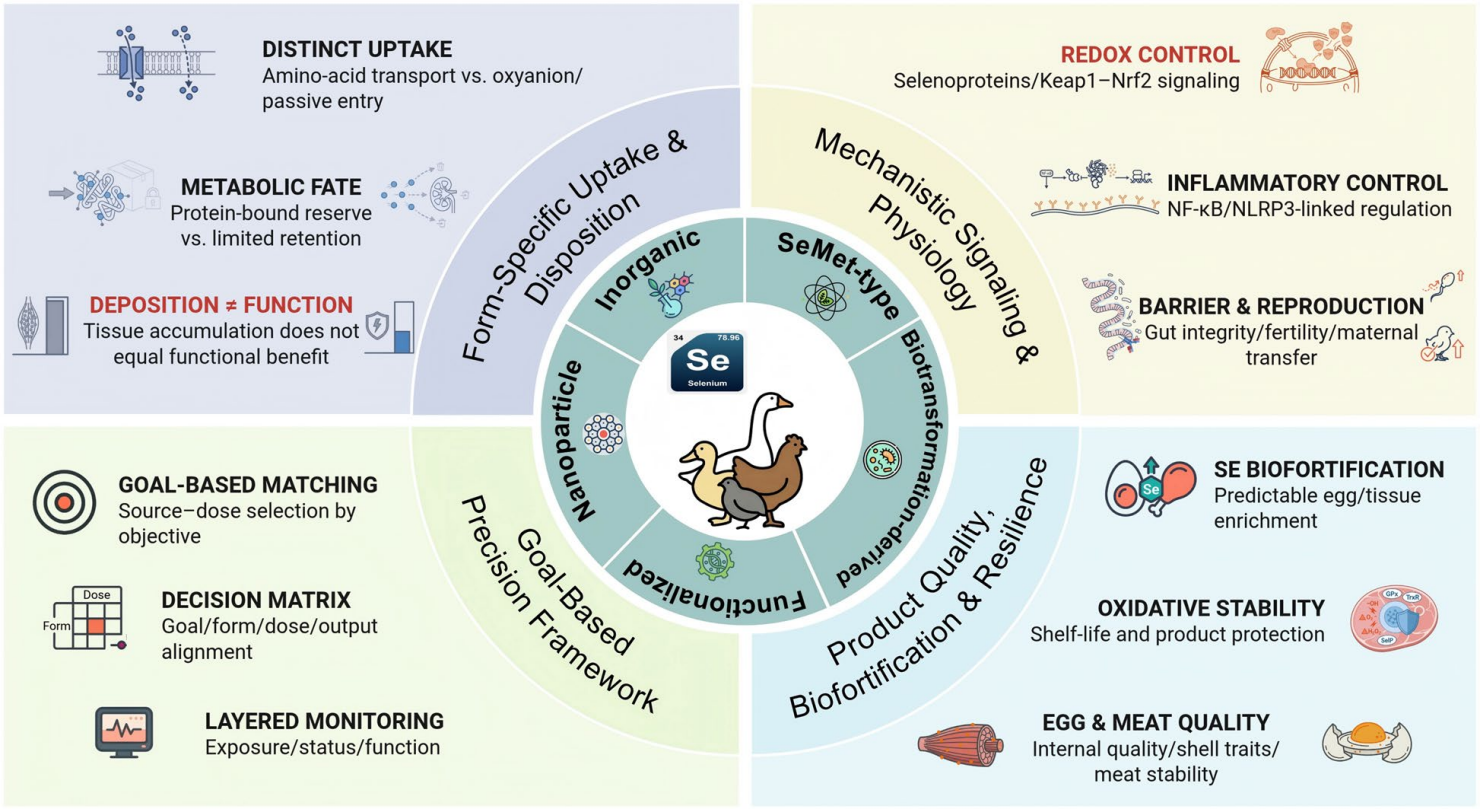

**Supplementary Information:**

The online version contains supplementary material available at 10.1186/s40104-026-01436-5.

## Introduction

### Background and objectives

Selenium (Se) serves as a critical determinant of physiological resilience in modern poultry nutrition. As the industry evolves toward antibiotic-free and high-efficiency production systems, birds face elevated metabolic demands and oxidative pressures. In this context, Se is no longer viewed merely as a micronutrient to prevent classical deficiency disorders (e.g., exudative diathesis or nutritional myopathy) [1]. Rather, it is recognized as a central regulator of redox homeostasis, immune competence, and endocrine stability [2–4]. This biological function is primarily mediated by a network of more than 20 selenoproteins, including glutathione peroxidases (GPx) and thioredoxin reductases (TrxR). These proteins coordinate peroxide control, thiol-redox cycling, and downstream cytoprotective programs to mitigate the physiological impact of rapid growth and environmental challenge [4, 5]. Consequently, optimizing Se status has become a strategic priority for flock health, production stability, and the biosafety of poultry products.

Total dietary Se alone, however, is an insufficient metric for comparing efficacy. Se bioefficacy is strongly influenced by chemical form, delivery matrix, and metabolic fate. Inorganic salts such as sodium selenite (SS) and selenate remain widely used because they are practical and cost-effective, yet their capacity to sustain reserve formation is limited in comparison with selenomethionine (SeMet)-type inputs [6–8]. By contrast, molecular SeMet-type sources can enter methionine-related metabolic routes and contribute to a protein-bound Se reserve that buffers fluctuations in demand or intake [8–10]. The picture becomes more complex with biotransformation-derived and engineered products. In Se-enriched yeasts (SeY) or microbial platforms, exposure is shaped by both Se speciation and biological matrix; in Se nanoparticles (SeNPs) and carrier-bound systems, physicochemical descriptors such as particle size distribution, coating identity, and colloidal stability become part of the exposure definition itself [9, 11–15]. Consequently, nominally matched Se doses do not necessarily represent biologically equivalent exposures, and comparisons across studies or products can be misleading when source identity is incompletely defined.

In recent years, the Se literature has rapidly matured from cataloging individual selenoprotein functions to embedding Se within stress-physiology and functional-food frameworks. Work in this period increasingly converges on signaling-level explanations. These developments focus especially on Se-dependent regulation of redox defense through the Keap1–Nrf2–ARE signaling pathway and immune/inflammatory control through NF-κB and NLRP3 inflammasome-linked pathways [14, 16–19]. However, despite this mechanistic progress, higher Se deposition, biomarker responses, and functional outcomes are still often discussed as though they were interchangeable indicators of efficacy, even though they do not necessarily reflect the same biological objective. As a result, the field still lacks frameworks that quantitatively link Se speciation and delivery inputs to biomarker responses and product-quality outcomes across diverse conditions [9, 20, 21]. This deficit is compounded by methodological limitations, specifically the lack of standardized biomarker panels and aligned endpoints, which precludes evidence-based selection of Se forms and dosages for targeted applications.

Against this backdrop, this review re-examines Se supplementation in poultry from a form-specific and application-oriented perspective. Specifically, it links source identity with mechanistic interpretation, physiological function, and implementation-relevant outcomes, with the aim of clarifying how Se form, exposure definition, and target endpoint should be considered together in more precise supplementation strategies for modern poultry systems.

### Review scope

As an integrative review, the scope prioritizes peer-reviewed primary research and high-quality reviews published primarily in the last six years (2020–2025). This timeframe reflects the rapid expansion of engineered and biotransformation-derived Se sources alongside the shift toward stress-physiology and functional-food frameworks. Early landmark papers were cited selectively when they remain essential for foundational mechanistic concepts or regulatory context (e.g., authorised maximum levels and classical tolerable-intake concepts).

Rather than aiming for exhaustive coverage, this review selectively organizes the literature around form-specific bioefficacy in poultry, with emphasis on how selenium source influences mechanistic interpretation and application-relevant outcomes. The purpose is to identify consistent patterns, clarify where conclusions remain source- or endpoint-dependent, and highlight the comparability gaps that continue to limit stronger precision guidance.

## Selenium forms, delivery strategies, and exposure window

### Chemical forms

**Fig. 1:**
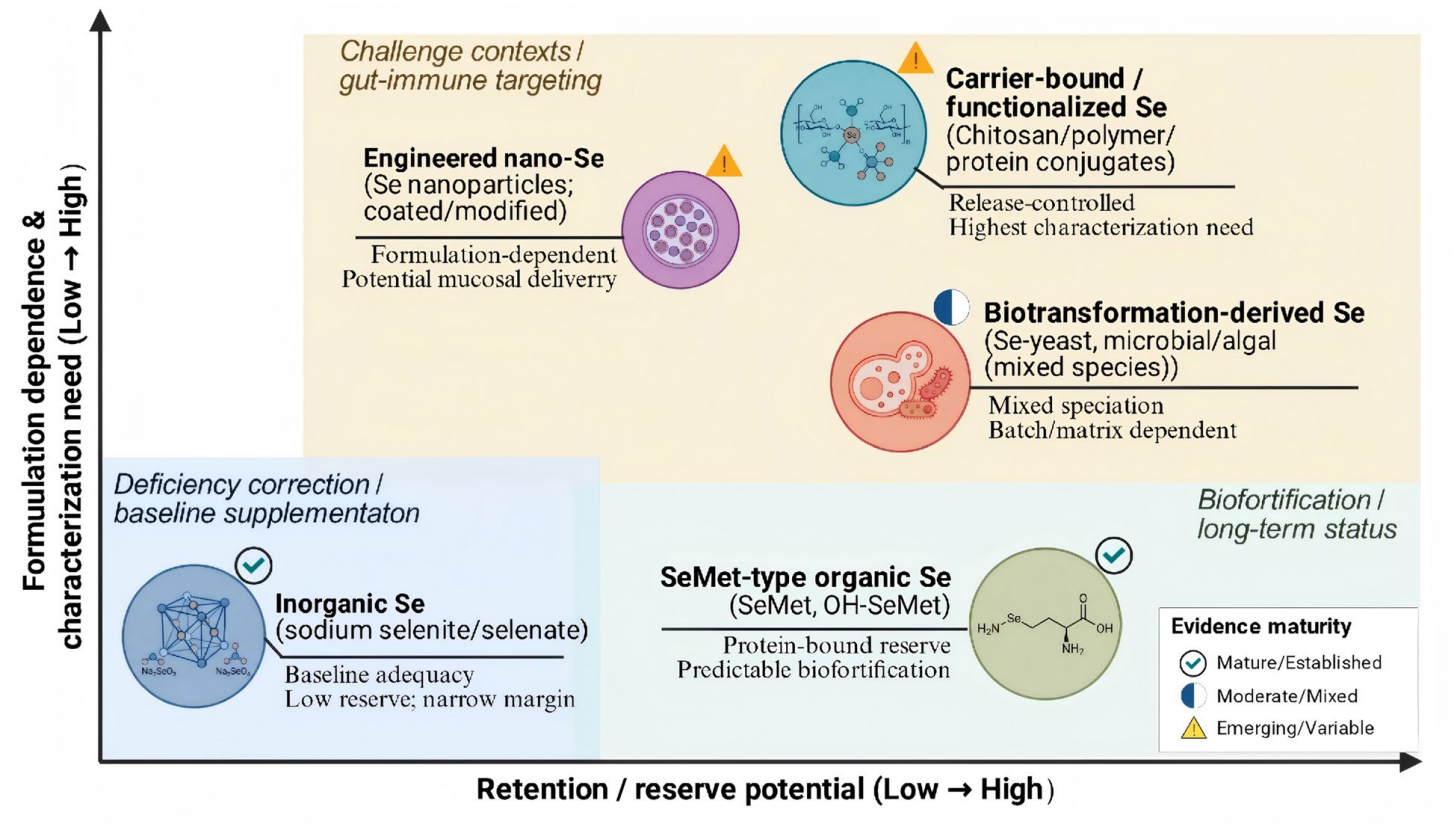
A typology map positioning major selenium source categories used in poultry by reserve-forming potential and formulation dependence. The *x*-axis represents the expected capacity to build and sustain body Se reserves, whereas the *y*-axis reflects the degree to which efficacy and comparability depend on specific formulation descriptors and characterization. Background shading highlights common application contexts. Symbols indicate evidence maturity based on the breadth and consistency of available data. These positions are conceptual and intended to summarize broad trends

The biological efficacy of Se in poultry varies substantially with its chemical form, because different Se species differ in intestinal uptake routes, conversion to the common selenide intermediate, and allocation between functional selenoprotein synthesis and reserve formation [8, 9, 20]. These form-specific kinetics influence both the predictability of tissue or egg enrichment and the stability of Se supply during stress. Accordingly, these source categories are useful not only for description, but also for interpretation, because they represent distinct exposure identities rather than interchangeable labels, ranging from defined inorganic oxyanions and molecular SeMet-type inputs to matrix-defined and formulation-defined systems. Given the narrow window between Se deficiency and excess, form selection is therefore central to balancing safety, efficacy, and production goals [2, 6, 22]. To orient the discussion, Fig. 1 positions major Se source categories according to reserve-forming potential and degree of formulation dependence. Table S1 provides a source-by-source comparison. In broad terms, inorganic Se is most suitable for adequacy-oriented use, SeMet-type molecular inputs are more relevant to reserve formation and predictable enrichment, whereas matrix-dependent sources require more product-specific interpretation.

#### Inorganic selenium

Inorganic Se, typically supplied as SS or selenate, represents the most clearly defined oxyanion-based exposure in poultry diets and remains the practical benchmark for meeting basal Se needs. Following ingestion, selenate is absorbed largely via sulfate co-transport systems, whereas selenite enters predominantly through non-carrier-mediated routes (primarily passive diffusion), after which both forms must be reduced to selenide (HSe^−^/Se^2−^), the common precursor for selenocysteine (Sec) synthesis and selenoprotein biosynthesis in the liver and other tissues [4, 5, 20]. Because inorganic oxyanions require sequential reduction steps before entering the selenoprotein pathway, their contributions to long-term retention and tissue deposition have been reported to be more variable than those of SeMet-type molecular sources under comparable dietary conditions [8, 23, 24].

Compared with Se-amino-acid–based sources, SS generally supports less efficient enrichment of edible fast-turnover tissues, although the tissue distribution pattern remains endpoint- and study-dependent [8, 23, 25]. Moreover, unlike SeMet-based sources that can be non-specifically incorporated into body proteins as a storage pool, selenite has limited capacity to build long-term protein-bound Se reserves, and excessive intake, particularly during heat stress or immune challenge, may exacerbate oxidative stress [9, 20, 26]. In practical terms, this profile aligns inorganic Se more closely with adequacy-oriented supplementation and deficiency correction, while making it less suitable for applications that rely on sustained reserve formation or highly efficient biofortification.

#### SeMet-type organic selenium

SeMet-type organic Se in poultry nutrition is commonly represented by molecular SeMet and OH-SeMet, both of which provide a relatively well-defined exposure compared with matrix-based products. A key mechanistic distinction is that these inputs share methionine transport and metabolic handling, enabling SeMet to be absorbed via amino-acid transporters and subsequently non-specifically incorporated into the body protein pool [4, 8, 27, 28]. This incorporation establishes a mobilizable, protein-bound Se reserve that can be gradually released through normal protein turnover, thereby stabilizing Se supply during prolonged challenges and improving the predictability of tissue/egg enrichment relative to inorganic salts. These advantages are most directly relevant to reserve formation, long-duration buffering, and predictable deposition, but do not by themselves establish universal superiority across all functional endpoints.

Among SeMet-type sources, OH-SeMet has been frequently used as a comparator in recent poultry studies and evaluated across multiple production-relevant stress contexts [10, 29–31]. In broilers under high-density heat stress, OH-SeMet improved feed conversion and increased tissue Se concentrations compared with SS, with additional improvements over SeY for selected redox/immune indices [10]. In post-peak laying hens, combining OH-SeMet with butyric glycerides improved laying performance and antioxidant readouts relative to SS [32]. These findings are consistent with the reserve-forming capacity of SeMet-type inputs and their ability to buffer fluctuations in Se availability when intake, utilization, or turnover is perturbed. Taken together, current poultry evidence most consistently supports SeMet-type advantages in deposition-related and stress-buffering outcomes, while evidence across a broader range of functional endpoints is still comparatively limited.

### Biotransformation-derived forms

Biotransformation-derived selenium products convert inorganic precursors into organic Se species within biological matrices, so exposure is defined by both speciation and carrier rather than a single pure molecule [9, 15, 33–38]. This category includes SeY, bacterial platforms (including probiotics), and algae, where Se may occur as SeMet and SeCys in proteins/peptides together with other Se species. As a consequence, bioefficacy can be shaped not only by Se chemistry but also by matrix-driven release, co-delivered bioactives, and batch-to-batch variability. Accordingly, SeY and related biotransformation-derived products should not be treated as single nutritional entities in cross-study comparisons.

Relative to inorganic salts, biotransformation-derived matrices can present Se in more nutrient-like contexts and co-deliver non-Se bioactives such as cell wall fractions, peptides, or pigments, thereby influencing both deposition patterns and functional outcomes in poultry [13, 39–44]. This potential advantage, however, should not be attributed to “organic Se” alone, because carrier bioactives and matrix-dependent release can materially shape the observed phenotype. SeY is the most established example within this category and has often been associated with higher retention and tissue or egg enrichment, plausibly because a substantial fraction of Se is present as SeMet embedded within a proteinaceous matrix that supports uptake and incorporation into tissue proteins [45–49]. At the same time, commercial SeY is chemically heterogeneous: reported SeMet proportions vary widely (e.g., 19%–72% of total Se), and other species such as residual elemental Se (Se^0^) and residual oxyanions can be substantial, with likely implications for bioefficacy, cross-study comparability, and batch-to-batch consistency [15]. Bacterial and algal platforms further broaden this category by combining Se delivery with carrier functionality or intrinsic nutrients [50–56]. For instance, Se-enriched *Bacillus subtilis* has been linked to increased body weight and beneficial shifts in gut microbial profiles in broilers [52]. Se-enriched lactobacilli similarly offer a probiotic alternative to yeast strategies, with reports of gut microbiota modulation and enhanced egg Se fortification in laying hens [13]. Algal platforms, such as *Chlorella vulgaris*, have also been explored, although their bioaccessibility remains highly species- and product-dependent [56, 57].

The major practical constraint on biotransformation-derived Se is not conceptual potential, but inter-product variability in both speciation and matrix composition. Robust characterization is therefore essential for meaningful comparison and informed additive selection, because differences in speciation and matrix composition can materially alter release behavior and bioefficacy [15]. Accordingly, biotransformation-derived Se is best viewed as a product-defined option whose value depends on adequate characterization and benchmarking against clear application goals rather than on category-level assumptions.

### Engineered selenium delivery systems

Engineered Se delivery systems utilize particulates or carrier-bound complexes to enhance stability and modulate gastrointestinal release, offering a more controlled alternative to simple salts or free seleno-amino acids [9, 58–60]. In contrast to biotransformation-derived matrices (where variability is driven largely by biological speciation and carrier composition), engineered systems are defined primarily by physicochemical descriptors and release behavior that determine mucosal interaction, uptake routes, and reproducibility. Accordingly, efficacy in this category is expected to be formulation-dependent, and cross-study comparability hinges on an exposure definition that includes both Se speciation and material descriptors. The conceptual novelty of this category lies in altered exposure geometry and mucosal interaction.

Nano-Se (typically SeNPs) is the most intensively studied engineered Se platform in poultry. Biological response strongly depends on formulation features such as particle size distribution, surface chemistry/coating, and colloidal stability, because these govern mucosal contact, dissolution behavior, and batch-to-batch consistency [21, 61–63]. Unlike SeMet-type sources, SeNPs may supply Se via a combination of particle-associated uptake and partial dissolution, which can shift distribution kinetics relative to molecular Se sources [58, 64–66]. Broiler trials have reported that SeNPs can support growth, Se retention, and health-related indices under certain conditions [11, 58, 66]. In one slope-ratio evaluation in broilers, the estimated relative bioavailability of the tested SeNPs preparation was lower than that of comparator sources (e.g., SeY, SeMet, and OH-SeMet), aligning with its limited capacity to build SeMet-type protein reserves relative to SeMet-type sources [8]. A recent dose–response meta-analysis in broilers likewise supported efficacy of nano-Se within a bounded inclusion range [21]. Toxicological interpretation should likewise remain formulation-specific, because SeNP surface chemistry, colloidal behavior, and physicochemical form can influence dissolution, tissue handling, and safety margins [67]. Therefore, current evidence supports nano-Se as an effective but formulation-dependent platform, especially for selected gut, redox, and challenge-related outcomes, rather than as a uniformly superior replacement for defined SeMet-type sources.

A notable development is carrier-assisted nano-Se, in which polymers or biopolymers are used to stabilize particles and tune mucosal interaction. More broadly, these systems show that, in engineered Se delivery, the carrier architecture is part of the exposure definition rather than a neutral background. For example, chitosan-loaded SeNPs have been reported to improve performance and intestinal morphology in broilers [60]. Such findings should be interpreted cautiously, because improvements in carrier-assisted systems may arise from carrier bioactivity and altered mucosal interaction as much as from the Se core itself [68, 69]. Beyond nanoparticles, polymer-bound and protein-based conjugates have also been explored as more controllable delivery formats, with some studies reporting improved laying performance, egg Se enrichment, or acceptable safety profiles [59, 70–75]. Overall, the translational value of these systems depends not simply on demonstrating efficacy, but on separating Se-core effects from carrier effects and on explicit reporting of source identity, material descriptors, and manufacturing consistency.

### Dose–response and safety thresholds

The Se dose–response in poultry is non-linear, often approximating a U-shaped relationship in which both deficiency and excess impair biological outcomes. However, a central reason for inconsistent conclusions in the selenium literature is that this response is often treated as a single curve, whereas different outcomes follow different response surfaces. The optimal range therefore varies not only with species, physiological stage, and exposure duration, but also with Se form, because different forms differ in absorption, retention kinetics, reserve behavior, and the extent to which they support functional pools versus storage [9, 20, 21, 76]. Accordingly, dose interpretation should be endpoint-specific and form-dependent.

Regulatory limits provide essential safety boundaries, but they should not be mistaken for functional optima or for evidence of equivalent biological response across sources. In the European Union, the authorized maximum total Se concentration in complete feed remains 0.5 mg/kg, with supplementation from organic sources limited to 0.2 mg/kg within that total, while the NRC (2005) maximum tolerable level for poultry is 3.0 mg Se/kg diet on a dry-matter basis [22, 77]. Within the commercially relevant range (approximately 0.15–0.45 mg Se/kg), increasing dietary Se generally raises tissue and egg Se concentrations, but the slope of deposition is form-dependent, consistent with source-specific differences in retention efficiency [8, 9]. These limits and response patterns indicate that dose interpretation must distinguish outer safety boundaries from biologically efficient dose ranges.

The most informative comparisons are those conducted at similar supplemental doses, because they show that source effects often diverge by endpoint even when nominal Se input is matched. At comparable supplemental levels (e.g., 0.2–0.25 mg Se/kg), nano-Se improved intestinal development and attenuated intestinal inflammation (NLRP3-linked responses) relative to other sources in young broilers, and enhanced performance together with antioxidant and immune indices under hot–humid stress compared with inorganic Se [12]. Yet under heat stress, nano-Se showed more limited protection of breast meat quality compared with other Se sources [26]. Such divergence indicates that source selection should be guided by the targeted endpoint rather than by deposition response alone, and that apparent efficacy depends on which biological layer is being assessed.

At supra-regulatory exposures, the distinction between deposition and function becomes even more important. SeMet-containing matrices can continue to elevate tissue and egg Se concentrations at multi-mg/kg levels, yet toxicity thresholds still emerge depending on form and dose. Evidence from laying quail (1.5–3.5 mg/kg) and hens (0.3–6.0 mg/kg) indicates that although SeY promotes dose-dependent Se deposition, excessive supplementation can precipitate overt pathological manifestations, notably reproductive attrition and hepatic impairment [47, 78]. Collectively, these data show that maximal deposition does not necessarily coincide with maximal functional benefit, and that many endpoints may reach diminishing returns before deposition plateaus. In precision terms, the relevant question is therefore not how far Se deposition can be increased, but which source–dose combination most efficiently improves the intended outcome within regulatory and toxicological constraints.

## Mechanistic modules of selenium bioefficacy

**Fig. 2:**
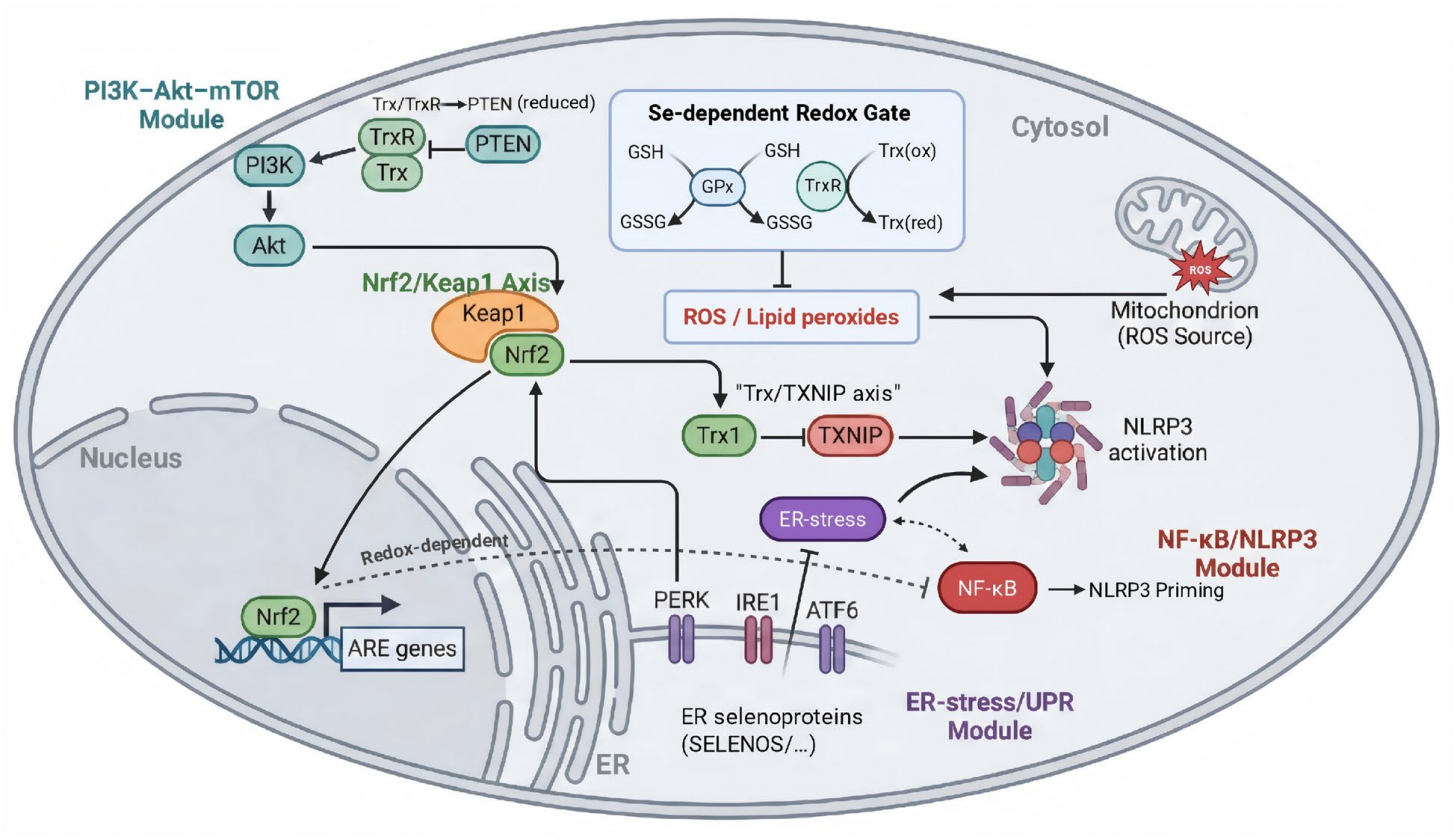
Mechanistic integration of Se-dependent redox homeostasis with inflammatory and metabolic signaling pathways. Dietary selenium supports key selenoproteins (GPx/GPx4, TrxR) that set cellular redox tone and limit ROS/lipid peroxidation, thereby coordinating cytoprotective Nrf2–ARE signaling and restraining NF-κB and NLRP3 inflammasome escalation. Central nodes include the Trx1–TXNIP axis and ER-stress/UPR modules (PERK/IRE1/ATF6), with additional tuning via redox-sensitive PTEN–PI3K–Akt coupling. Solid arrows, blunt-ended lines, and dashed arrows indicate activation, inhibition, and indirect/context-dependent crosstalk, respectively

Se bioefficacy in poultry can be interpreted through a linked sequence of mechanistic modules. Se form shapes exposure kinetics to the selenide pool and reserve stability, which together influence selenoprotein redox capacity via GPx and TrxR. This redox gate regulates cellular ROS tone and downstream redox-sensitive signaling responses. Figure 2 provides an integrated view of the central coupling points across these pathways.

### Upstream control of selenoprotein output

Dietary Se exerts its bioefficacy primarily by supplying the intracellular selenide pool, the common metabolic entry point for Sec synthesis and subsequent selenoprotein production. Although inorganic, organic, biotransformation-derived, and engineered Se inputs ultimately converge on this pool, Se form determines how efficiently and how stably Se is delivered to it, particularly when intake fluctuates or physiological demand increases. Inorganic oxyanions (typically SS) require sequential reduction before contributing to selenide, which can constrain flux under stress and provides limited opportunity to build durable body reserves [2, 79]. By contrast, SeMet-type molecular inputs (SeMet and OH-SeMet) can be converted to selenide when required and can also be incorporated non-specifically into the body protein pool in place of methionine, creating a mobilizable, protein-bound reserve that releases Se gradually during routine protein turnover [4, 23, 80]. This reservoir-like behavior provides a mechanistic basis for steadier Sec availability during prolonged challenge and helps explain why distinct Se forms can diverge even before downstream signaling pathways are considered.

At the molecular level, selenoprotein biosynthesis depends on selenophosphate generation and a specialized Sec insertion sequence (SECIS)-dependent translation system [81, 82]. In poultry, emerging evidence indicates that the Se source can modulate not only end-product selenoproteins but also upstream biosynthetic components. For example, a bacterial Se source (*Stenotrophomonas maltophilia*, ADS18) up-regulated mRNA expression of *GPX1*, *GPX4*, *DIO1*, *DIO2*, and *SELW1* more effectively than SS [83]. Se-enriched lactobacilli up-regulate genes involved in Se recycling and utilization, including *SEPHS1*, *SCLY*, and *TXNRD1* [13]. In early-life programming, highly bioavailable Se provided via in ovo or maternal strategies has also been reported to prime later selenoprotein expression, suggesting that timing and exposure form can shape longer-term biosynthetic responsiveness [84, 85]. In summary, these findings indicate that Se form and delivery matrix influence not only the final activity of individual selenoproteins, but also how efficiently and how stably birds can sustain selenoprotein synthesis. In practical terms, the key difference is that all Se sources converge on the same Sec biosynthetic pathway, but they do not supply it with equal speed, persistence, or buffering capacity.

### Redox gating and adaptive defense programs

Redox control is the most consistently mapped mechanistic module of Se bioefficacy in poultry, but the central issue is regulation of redox signaling capacity rather than a generalized rise in antioxidant status. Two coupled components are particularly important. The first is an enzymatic layer, in which Sec supports core antioxidant selenoenzymes, most notably GPx/GPx4 and TrxR, that constrain peroxide and lipid-peroxide tone and maintain thiol redox cycling within and across cellular compartments [5, 21, 86, 87]. The second is an adaptive transcriptional layer, in which redox tone feeds into programs such as Keap1–Nrf2–ARE signaling that coordinate phase-II detoxification and broader cellular defense networks [19, 88, 89]. Under matched experimental conditions, organic Se molecular inputs and biotransformation-derived matrices have often been associated with stronger engagement of Nrf2-linked transcriptional readouts than inorganic salts, consistent with more stable intracellular Se supply to Sec-dependent redox systems [19, 87]. Dose–response relationships are also frequently non-linear: moderate supplementation can enhance GPx/TrxR capacity and Nrf2-linked gene expression, whereas excessive exposure may blunt adaptive signaling or promote pro-oxidant stress, with risk profiles shaped by form and exposure kinetics [6, 20, 27].

Se source differences in redox control are commonly explained by delivery kinetics and reserve buffering. SeMet-type molecular inputs tend to support more sustained redox capacity because protein-pool buffering stabilizes substrate availability for continued selenoprotein synthesis [5, 23, 80]. Conversely, SeNPs may trigger more rapid cellular redox responses through particle-associated uptake and partial dissolution, but generally show weaker capacity to support long-term SeMet-like reserve dynamics [61, 90]. Redox control also interfaces with broader cytoprotective programs. In poultry, Nrf2-linked responses and selenoenzyme systems can couple to PI3K–Akt signaling, linking nutrient status with cytoprotection and repair programs [86, 91–94]. Consequently, different Se sources may show comparable GPx activity yet diverge in transcriptional adaptation and downstream protection outcomes. This helps explain why source effects are often more discriminating under oxidative or inflammatory challenge, when adaptive redox signaling becomes rate-limiting [88, 95]. Overall, Se-dependent redox control integrates enzymatic capacity (GPx/TrxR) with adaptive signaling (Keap1–Nrf2–ARE), and apparent advantages are endpoint- and context-dependent rather than universally superior.

### Redox-linked inflammatory signaling

In poultry, Se influences immune regulation largely through redox-sensitive inflammatory thresholds rather than directly suppressing cytokine output. By shaping selenoprotein-dependent redox capacity and redox-responsive transcriptional programs, Se can bias inflammatory set points during challenge [3, 4, 20]. In detail, the most informative framework is the coupled NF-κB/NLRP3 module, which can be parsed into priming and activation steps. NF-κB signaling is central to the priming phase, in which TLR engagement and adaptor signaling (MyD88/TRIF) drive transcription of pro-inflammatory programs and increase the availability of inflammasome components [96]. In contrast, the NLRP3 inflammasome represents an activation layer that governs caspase-1-dependent cytokine maturation and inflammatory amplification [97]. As summarized in Fig. 2, the Trx1–TXNIP axis provides a key redox–inflammasome coupling node, linking changes in thiol redox state to inflammasome activation thresholds [98].

For comparisons among Se sources, pathway-level readouts are often more informative than distal immune phenotypes, because they more directly capture how Se inputs reset inflammatory signaling thresholds. Poultry evidence suggests that Se form can shift these set points, especially when Se is delivered in carrier-containing matrices that also provide biological cues. For instance, Se-enriched probiotics have been associated with altered expression of innate immune receptors and antimicrobial programs [51–53, 99]. This pattern is consistent with a model in which the carrier contributes immune cues while Se helps restrain excessive inflammatory escalation. Engineered formats show related inflammasome-level effects: SeNPs have been reported to reduce NLRP3-related signals and downstream inflammatory mediators while supporting epithelial or immune-cell resilience [12]. Across challenge models, cytokine profiles often shift in a source-dependent manner, but the direction and magnitude of these shifts remain contingent on baseline Se status, stressor type, and dose [100–103]. Accordingly, measures such as NF-κB activation state, TLR adaptor expression, and inflammasome components provide a more discriminating basis for mechanistic comparison than distal immune phenotypes alone [12, 103, 104]. Overall, Se appears to modulate poultry immunity through form- and context-dependent tuning of NF-κB priming and NLRP3 inflammasome activation within a redox-regulated inflammatory module.

### Cell stress and metabolic programming

Beyond redox buffering and inflammatory threshold setting, Se also influences downstream stress-response programs that determine whether challenged tissues sustain repair, preserve proteostasis, or progress toward dysfunction. In this sense, PI3K–Akt-linked survival signaling, endoplasmic reticulum (ER)-stress, unfolded protein response (UPR) capacity, and cell-fate routing can be viewed as distal consequence layers of earlier redox and inflammatory divergence.

Within this framework, PI3K–Akt–mTOR signaling represents a repair and survival module through which Se status can bias growth or recovery capacity. In broiler myoblasts, Se promoted proliferation through the ROS/PTEN/PI3K/Akt axis, indicating redox-sensitive coupling between nutrient status and repair signaling [91]. Conversely, Se deficiency in chicken spleen has been linked to growth restriction together with reduced IGF-1R/PI3K/Akt/mTOR signaling, consistent with impaired pro-survival programming under low Se status [105]. These findings support PI3K–Akt as a vital integration node through which Se status can bias repair capacity during challenge. Se also intersects with proteostasis capacity: Se deficiency has been associated with activation of ER-stress signaling in immune tissues [106], whereas in a broiler ascites model SeNPs suppressed UPR-related signals (e.g., *GRP78*/*ATF6*/*CHOP*-linked readouts) together with downstream apoptosis mediators [107]. Under more sustained pressure, these effects converge on cell-fate routing through autophagy, apoptosis, and mitophagy, as illustrated in pancreatic injury models where low Se status coincided with mitochondrial dysfunction and altered mitophagy/apoptosis markers alongside shifts in PTEN/PI3K/Akt/mTOR signaling [92]. Across such contexts, form effects are plausibly time-scale dependent: engineered inputs may alter short-term intracellular availability, whereas SeMet-type inputs can maintain longer-duration support via protein-pool buffering when prolonged supply stability is limiting [8, 9]. Overall, this module suggests that Se sources diverge because they differ in how persistently they support intracellular repair, proteostasis, and cell-fate control under challenge.

## Physiological function of selenium in poultry

### Antioxidant defense and redox homeostasis

In poultry, Se is a central contributor to antioxidant defense and redox homeostasis. This is manifested by enhanced control over lipid peroxidation and the preservation of redox-sensitive tissue integrity during nutritional or environmental stress. Common readouts include higher GPx activity and total antioxidant capacity (TAC), together with lower malondialdehyde (MDA) or related lipid-oxidation indices in key tissues [87, 108, 109]. These antioxidant-related outcomes are more accurately interpreted as redox buffering capacity, that is, the ability to stabilize peroxide tone during challenges like heat stress, immune activation, or toxin exposure.

Form-dependent advantages become most visible when birds are exposed to supra-basal oxidative pressure. At equivalent inclusion levels, SeMet-type molecular inputs (SeMet and OH-SeMet) and SeY are often reported to better sustain redox homeostasis by maintaining Se availability via protein-pool buffering [2, 9]. For example, in broilers under combined high stocking density and heat stress, OH-SeMet improved systemic Se status, increased GPx activity and TAC, and reduced MDA and protein carbonyl in muscle compared with SS [10]. Inorganic selenite remains effective for correcting deficiency and supporting basic antioxidant function, but its limited reserve formation can translate into greater variability in tissue redox control when demand rises or intake fluctuates.

At the same time, redox benefit does not always appear as a single, uniform pattern across endpoints. In laying hens supplemented with 0.3 mg Se/kg, SeNPs and SeY both increased serum TAC, but SeY more strongly elevated catalase and superoxide dismutase (SOD) activity, whereas SeNPs more clearly reduced serum MDA levels [108]. Notably, hepatic antioxidant gene mRNA changed little among groups, indicating that Se can improve functional redox status through different regulatory layers, including enzyme activity and damage containment, without necessarily producing large transcriptional shifts in liver. Taken together, antioxidant endpoints discriminate Se sources most clearly under supra-basal oxidative pressure: sources with reserve-forming capacity tend to provide steadier redox buffering during sustained challenge, whereas differences under basal conditions are often modest and endpoint-specific.

### Systemic and mucosal immunity

In poultry, Se supports immunity by modulating redox-sensitive inflammatory signaling while preserving effective host defense. At the systemic level, this is often reflected by improved immune-organ status and a cytokine profile that limits excessive pro-inflammatory drift during challenge while maintaining functional Th1-type responsiveness (e.g., IFN-γ, IL-2) [100–102]. At mucosal surfaces, Se adequacy matters because the gut and respiratory tract are continuously exposed to environmental antigens and depend on controlled inflammatory tone to avoid tissue-damaging immune escalation. Practical readouts include secretory IgA (sIgA), local cytokines and antimicrobial programs, alongside reduced mucosal injury [14, 84]. Barrier- and microbiota-centered outcomes are discussed separately in Sect. “Gut barrier function and microbiota”.

In poultry practice, Se functions less as a non-specific immune stimulant than as a modulator that improves coordination between systemic and mucosal defense. In vaccinated broilers, selenised garlic polysaccharides increased systemic hemagglutination-inhibition antibody titers together with jejunal and tracheal mediators (sIgA, IL-2, IFN-γ), demonstrating improved systemic–mucosal coupling under antigen exposure [110]. Under toxicological stress, dietary SS restored cecal-tonsil humoral capacity in aflatoxin B_1_ (AFB_1_)-challenged broilers, with higher IgA^+^ cell counts and up-regulated *IgA*, *IgM*, *IgG*, and *pIgR* mRNA expression, consistent with recovery of local mucosal defense alongside broader humoral competence [111].

Se inputs can nevertheless produce distinct immunological signatures dictated by form and delivery context. In laying hens, SeMet showed a clearer dose–response in antibody-associated indices, with 0.15 mg/kg producing higher serum IgA/IgM and duodenal/jejunal sIgA levels than 0.30 mg/kg under standard conditions [80]. By contrast, carrier-containing systems may shift innate or mucosal readouts in ways that are more difficult to attribute to Se chemistry alone. For example, *Chlorella vulgaris* combined with SeNPs increased phagocytic activity and reshaped cytokine profiles in broilers [112], while Se-polysaccharide complexes have shown coordinated adaptive–mucosal outputs [110, 113]. Collectively, Se appears to support poultry immunity more consistently by limiting maladaptive inflammatory drift and preserving coordinated systemic–mucosal defense than by uniformly amplifying immune activity; positive findings in carrier-containing systems should therefore be interpreted as formulation- and context-dependent rather than attributed to Se chemistry alone.

### Gut barrier function and microbiota

Se contributes to gut resilience largely by supporting selenoprotein-dependent antioxidant defense and epithelial renewal, which helps stabilize structure, limit barrier leak, and dampen inflammation [12, 14, 114]. Consistent with this, supplementation studies frequently report improved villus morphology, stronger tight-junction expression, better goblet-cell function and a more favorable microbial profile, although the magnitude varies with Se source and context [12, 52, 84, 115].

Gut outcomes often combine structural and ecological shifts. In aged laying hens, SeY induced broad ileal reprogramming with concurrent remodeling of microbial communities toward *Lactobacillus*- and *Veillonella*-enriched profiles while suppressing opportunistic taxa, aligning metabolic adaptation with improved redox and inflammatory tone [116]. Microbial-derived Se can further add a carrier-plus-Se effect, as shown by reports of improved villus development, secretory IgA, and barrier-associated microbiota patterns in Se-enriched *Bacillus subtilis* or lactobacilli systems [13, 51].

Engineered systems provide further leverage at the mucosal interface [115]. Green-synthesized SeNPs and chitosan-loaded SeNPs have been associated with larger villus surface area, increased acidic goblet cells and shifts toward higher *Lactobacillus* with lower *Escherichia coli* [60, 66]. Maternal supplementation with nano-Se improved offspring jejunal villus structure, tight-junction transcripts, goblet cells and Muc2 in day-old chicks [84]. Overall, the most informative assessment of Se effects on gut health integrates epithelial architecture, junction- or mucus-related readouts, and microbial ecology under defined conditions rather than relying on any single endpoint. More pronounced benefits in microbial or engineered delivery systems should therefore be interpreted cautiously, because they may reflect advantages in mucosal delivery context as much as differences in Se chemistry itself.

### Reproductive function and maternal transfer

Reproductive function is among the most Se-sensitive physiological domains in poultry because gametes and embryos are highly vulnerable to lipid peroxidation and redox imbalance. Functionally, Se contributes to (i) male fertility through sperm viability, motility and membrane stability, (ii) female reproductive competence through follicle or oviduct function and endocrine support, and (iii) maternal transfer into egg compartments that sustain embryonic development. Accordingly, reproductive efficacy is commonly evaluated by semen quality, fertility and hatchability, and early progeny robustness [29, 45, 117–124]. Concurrently, controlled in ovo Se delivery has emerged as a useful experimental strategy for probing early-life programming of antioxidant status, intestinal immunity, and later performance, although it should not be treated as a direct equivalent of routine feed supplementation [125–129].

Maternal transfer studies in breeder ducks illustrate how moderate Se can translate into reproductive success. Supplementing 0.27 mg Se/kg SS maximized fertility and hatchability while increasing offspring antioxidant enzyme activity (e.g., GPx3, GPx1), consistent with effective transfer of redox protection to the next generation [119]. Synergistic SS plus iodine (0.2 mg Se/kg + 0.4 mg I/kg) further improved hatchability and progeny development indices, increased egg-yolk Se deposition, and was associated with up-regulation of pathways linked to antioxidant and growth regulation (e.g., Nrf2, SHMT1, IGF-1) [120]. Consistent with this maternal-transfer framework, duck studies also report heavier hatchlings and improved offspring antioxidant status following maternal Se supplementation [130].

Organic Se sources show clear benefits in both sexes through functional endpoints that map onto redox and endocrine stability. In roosters, SeMet at 0.5 mg/kg optimized testicular antioxidant status (T-SOD, CAT, GPx), reduced oxidative damage (lower MDA), and increased GPx4, a key determinant of sperm integrity [118]. In geese, SeY improved sperm kinematic parameters and testicular histology, and in females it yielded the highest egg fertilization rate, and SeY combined with zinc lysine further improved reproductive hormones and germ-cell indices [121]. Notably, typical supplementation rarely alters gross reproductive morphology. Neither SS nor SeMet changed ovary weight, follicle numbers or oviduct dimensions [131], and Se–insect protein complexes likewise did not alter organ indices despite signaling changes [74]. Taken together, the reproductive value of Se is expressed mainly through functional and transferable outcomes, including improved semen quality, fertility or hatchability, and embryonic or early-life robustness, mainly supported by improved antioxidant capacity in reproductive tissues and in offspring.

## Production and implementation

### Performance and stress resilience

In commercial production settings, Se contributes to performance mainly by reducing stress-induced penalties, rather than by pushing growth beyond genetic limits under ideal conditions. This shows up most clearly in heat load, high-density, toxin exposure, and other oxidative and inflammatory pressures. Meta-analytical evidence confirms this paradigm, demonstrating that while Se supplementation consistently improves antioxidant status and productive traits in heat-stressed broilers, the magnitude of these benefits is fundamentally amplified by the severity of the environmental challenge [25].

When oxidative pressure is sustained, SeMet-type inputs are often more effective at maintaining flock performance because they support whole-body Se status and functional antioxidant capacity more consistently over time [10, 132, 133]. In broilers subjected to combined heat and density stress, OH-SeMet (0.3 mg Se/kg) outperformed SS and SeY in improving feed conversion ratios, accompanied by superior tissue Se retention and reduced systemic stress markers (e.g., lower cortisol and inflammatory indicators) [10]. This resilience is consistent with a broader requirement for stable Se supply under prolonged thermal stress. Recent studies in tropical production environments likewise indicate that performance under heat load depends not simply on Se inclusion per se, but on source choice and its capacity to sustain bioavailable Se over time [132].

This resilience extends to toxicological challenges, where Se acts as a critical constraint-lifter against oxidative injury. In an AFB_1_ challenge model in Japanese quail, Se supplementation, particularly SeNPs at 0.5 mg/kg, partially reverses toxin-induced depressions in feed intake and weight gain while enhancing TrxR activity and antibody responses [134]. Under cadmium exposure, Se also mitigated stress-related injury by suppressing toxic metal accumulation and restoring essential element profiles in pectoral muscle, thereby preserving muscle nutritional integrity under heavy-metal stress [135]. However, the efficacy of novel Se forms remains context-dependent. Recent comparative studies indicate that while most Se sources alleviate heat-associated disruptions, certain nano-formulations may show weaker protection of metabolic and breast-muscle readouts than organic forms [136, 137]. In practice, Se supports production most consistently by limiting stress-related performance losses; reserve-forming sources are often more reliable under sustained multi-stressor pressure, whereas apparent advantages of nano or other novel systems remain highly contingent on formulation and the endpoints prioritized.

### Meat quality and shelf-life

Poultry meat quality, encompassing tenderness, juiciness, color, water-holding capacity (WHC), and oxidative stability, is a critical determinant of consumer acceptance and product shelf-life [138–140]. A recent mini-review specifically summarizes selenium utilization in broiler skeletal muscle and links selenoprotein-mediated antioxidant function to meat-quality regulation and prevention of muscle injury [141]. Across studies, Se improves meat quality mainly by limiting postmortem oxidative deterioration and preserving myofibrillar and mitochondrial ultrastructure, but the magnitude and pattern depend on Se form, diet context, and tissue fatness. However, increased tissue Se deposition does not necessarily translate into improved eating quality. In practice, efficacy is better evaluated by the extent to which lipid and protein oxidation are restrained during storage and processing.

Comparative trials suggest that SS can effectively raise tissue Se concentrations, but in some settings it has been associated with less favorable technological traits, such as reduced WHC, unfavorable color shifts, and ultrastructural changes (e.g., reduced sarcomere length or myofibrillar disruption), that may contribute to poorer tenderness or oxidative stability [142, 143]. Organic sources often perform better on oxidation-linked endpoints. In some studies, SeY has been reported to reduce lipid oxidation and improve tenderness relative to SS at comparable inclusion levels [24]. This aligns with reports linking SeY to improved color stability and oxidative resistance, although outcomes remain diet- and context-dependent [23]. Notably, while SeMet-type inputs achieve high deposition, their impact on conventional meat quality can be limited by extrinsic factors such as dietary fat, storage conditions or slaughter stress [142].

Engineered forms such as nano-Se and carrier-assisted systems can further improve specific quality attributes when well-formulated. Reports describe reduced drip loss, more stable pH, improved color, and better WHC at practical inclusion levels, together with meaningful Se enrichment of edible muscle [11, 143]. Robust shelf-life benefits may be further enhanced in synergistic combinations, where Se is paired with other bioactive compounds. Examples include SeY combined with jujube powder, Se-enriched *Cardamine violifolia*, or Se-phytobiotic complexes, which collectively improve redness, reduce cooking losses, and suppress oxidative markers (e.g., TBARS/MDA and protein carbonyls), particularly in thigh muscle where the risk of lipid oxidation is higher [144–147]. Notably, emerging metabolomics studies have linked SeNP-mediated improvements in meat quality under heat stress to ferroptosis-related regulation [148]. Overall, for meat applications, the decisive criterion is oxidative and structural stability during storage and processing; in this context, organic or well-formulated combined systems often outperform inorganic Se, although the advantage remains strongly shaped by tissue type and post-slaughter conditions.

### Egg quality and selenium biofortification

Modern layer nutrition has transitioned from meeting basal Se requirements toward the precision design of eggs with predictable Se enrichment and stable quality. A critical production distinction is separating deposition efficiency (how rapidly and how much Se transfers into yolk/albumen) from quality preservation (how well internal and shell traits resist storage- and age-related deterioration). Functionally, egg-oriented applications should therefore be judged on two related but distinct axes: controlled Se transfer into yolk and albumen, and maintenance of internal and shell quality under commercial and aging conditions.

SeY remains a reference strategy for egg Se enrichment due to its predictable enrichment kinetics, which facilitates tiered production. Its dose–response behavior is often reported as linear or quadratic, supporting practical formulation. For example, inclusion levels around 0.1 mg/kg meet standard table-egg goals, whereas inclusion up to 0.4 mg/kg is typical for biofortified eggs [131]. Kinetics are also production-relevant. In ducks, 0.25 mg/kg SeY increased whole-egg Se within two weeks, enabling planned enrichment windows [149]. Comparative trials further indicate that SeY enriches eggs more efficiently than inorganic sources and certain nano-Se preparations over the same period [108, 150].

A second key point is that maximizing deposition is not the same as optimizing egg quality. High dietary Se (reported up to 6 mg/kg) can continuously increase egg Se content. However, internal quality traits such as Haugh units and yolk color tend to plateau at moderate inclusion levels of approximately 1.5 mg/kg [13, 47]. Beyond deposition itself, SeY can contribute to quality-relevant functionality by reshaping egg proteins across albumen and yolk, thereby modifying albumen functional or digestive properties and the profile of Se-containing yolk proteins, consistent with enhanced generation of low-molecular-weight antioxidant peptides after digestion [151, 152]. In aging hens, SeY has also been linked with improved shell quality via uterine transcriptional programs associated with mineralization [45]. Some newer carrier-based systems may provide supplementary benefits [37, 38, 153, 154], but current evidence remains more product-specific than for SeY-based enrichment strategies. Taken together, for egg-oriented applications, Se programs should prioritize sources with predictable transfer kinetics rather than maximal deposition per se; enrichment and quality preservation should therefore be evaluated separately rather than assumed to peak at the same dose.

## Precision application and future directions

### Matching form and dose to defined goals

Precision Se nutrition begins by rejecting three common simplifications: total dietary Se is not itself an efficacy metric; matched nominal doses do not ensure matched biological exposure; and maximal deposition is not equivalent to maximal functional benefit. In practice, Se programs should first define the primary objective—baseline adequacy, stress resilience, barrier competence, or targeted egg/meat enrichment—and only then align source, dose, and timing to that goal.

For baseline adequacy, clearly defined inorganic sources remain practical and defensible, particularly in cost-sensitive programs [20, 23, 24]. However, when the goal is prolonged stress buffering or more predictable tissue/egg transfer, the best-supported first choice is usually a reserve-forming SeMet-type input, because its protein-pool buffering stabilizes Se supply over time [10, 108, 131, 149]. This distinction becomes most evident under challenge. A meta-analysis of 74 trials in heat-stressed broilers showed consistent improvements in antioxidant status and productive performance, but the magnitude of response depended heavily on heat-stress severity, Se source, dose, and tissue sampled [25]. Comparative studies further suggest that, even at matched nominal inclusion, SeY or SeMet-type inputs can outperform some nano-Se formulations for selected breast-muscle and selenoprotein-expression endpoints under heat stress [136].

For biofortification goals, predictable transfer kinetics are often more informative than maximal deposition per se. SeY remains the most consistently supported reference when graded and reproducible egg enrichment is required [47, 108, 131]. However, quality preservation still requires separate verification, since deposition and internal-quality traits do not necessarily peak at the same dose [13, 47]. Microbial or engineered systems may be justified when the intended advantage involves mucosal delivery or barrier-related outcomes, but these options should be selected only when the product is sufficiently defined and its benefit is demonstrated in challenge-relevant models rather than assumed from source category alone [12, 13, 51, 114]. Therefore, form and dose should be specified as a paired, goal-based strategy and evaluated against outcome-relevant endpoints rather than total Se alone. Table 1 summarizes this decision logic.

**Table 1:** Goal-based decision matrix

**Production goal**	**Candidate Se form(s)**	**Common study range (Se-equivalent)**^**a**^	**Exposure (verify intake)**	**Status (Se biology)**	**Function (fit to goal)**	**Representative references**
Baseline adequacy; correct deficiency	SS (first-line); low-dose SeY	~ 0.15–0.30 mg Se/kg feed	Analyzed total Se; feed intake; Se intake	Plasma/whole-blood Se; blood GPx	Growth/lay performance; mortality; basic oxidative marker	[77, 155, 156]
Egg/meat Se biofortification	SeY; OH-SeMet; SeMet	~ 0.10–0.40 mg Se/kg feed	Analyzed total Se; egg/meat Se output	Plasma or tissue Se; SelP/GPx as supportive markers	Egg/meat Se concentration; quality preservation if claimed	[8, 108, 131, 149]
Stress resilience (heat/high density/multi-stressor)	OH-SeMet; SeY	~ 0.15–0.35 mg Se/kg feed	Analyzed diet Se; intake	GPx/TAC; MDA/TBARS; key cytokines	FCR/ADG or egg mass; survival; stress indices	[10, 31, 132, 136]
Gut barrier/mucosal support	Product-defined microbial Se carriers; selected engineered Se systems	~ 0.20–0.40 mg Se/kg feed	Analyzed total Se; product identity; intestinal Se (optional)	Intestinal GPx; inflammatory signaling (e.g., NF-κB/NLRP3)	Villus/crypt; tight junction markers; microbiota; diarrhea/lesion scores	[12, 51, 114]
Late-lay/aging support	SeY-supported or plant-enriched product-defined systems (emerging evidence)	~ 0.30 mg Se/kg feed in current studies; context-dependent	Analyzed total Se	Liver antioxidant and lipid markers	Hepatic fat; egg quality; storage oxidative stability	[37, 38, 116]
Reproduction/hatchability/progeny robustness (feed-based)	SeMet-type; mixed SS + organic Se	~ 0.10–0.30 mg Se/kg feed	Analyzed total Se; egg/yolk Se as transfer indicator	Parental GPx/SelP; embryo antioxidant markers	Fertility; hatchability; embryo mortality; progeny robustness	[119, 120, 122]
In ovo Se delivery (experimental programming model)	Selenite; organic Se; LAB-derived nano-Se	1.5–30 µg/egg	Delivered dose; form; route/timing	Embryo antioxidant markers; immune readouts; liver/intestine morphology	Hatchability; early growth/FCR; gut/immune traits	[125, 127, 128]
Safety-window boundary (research only)	Any form; interpret as safety/boundary testing	> 1.0 mg Se/kg feed (multi-mg/kg)	Verified diet Se; intake; tissue Se	Liver enzymes; histopathology; oxidative damage markers	Performance plateau vs adverse effects; organ toxicity	[6, 22, 47, 77]

^a^Indicative ranges reported in the poultry literature; not regulatory recommendations. Verify total Se analytically in finished feed and comply with local maximum permitted levels and product-specific Se-equivalency

### A layered monitoring framework: exposure, status, and function

Operational precision requires separate monitoring of exposure, physiological status, and functional efficacy, because single markers (especially GPx alone) can obscure form- or context-specific differences. Exposure monitoring should begin with source verification beyond total Se quantification. For biotransformation-derived products, minimum reporting should include the major organic species and residual non-organic fractions (e.g., SeMet proportion, Se^0^, and residual oxyanions where available); for engineered products, key physicochemical descriptors such as particle size distribution, surface chemistry/coating, and colloidal stability are essential. Without this information, matched-dose comparisons are not interpretable across studies or farms. Table S2 summarizes a minimum comparability set for each source category.

Status monitoring should then assess whether Se supply is adequate for the relevant tissues rather than assume a single universal saturation point. Plasma Se and whole-blood GPx remain useful metrics but should be interpreted within the context of tissue priorities and response ceilings. A more informative framework anchors adequacy to broader selenoproteome behavior rather than single enzyme activity. In broilers, optimal doses estimated from selenoprotein-expression profiles have been reported at approximately 0.36 mg/kg for the liver and 0.46 mg/kg for the pancreas [155], underscoring that physiological adequacy is tissue-specific. Systems-level signatures, such as coordinated suppression of PI3K/Akt/mTOR signaling under Se deficiency [157], further support integrated status assessment.

Functional monitoring should pair capacity markers (e.g., GPx/TrxR-related readouts) with damage markers (e.g., MDA/TBARS), and include outcome-relevant compartments (e.g., egg/meat Se enrichment), aligning with the endpoints currently utilized in informative production meta-analyses [25]. This layered design is vital to operationalizing precision Se nutrition, because functional success cannot be inferred from exposure or status markers alone.

### Limitations and future perspectives

The main barrier to stronger precision guidance is comparability. For biotransformation-derived products, matrix and speciation heterogeneity are major confounders. Large variations in SeMet and Se^0^ content across SeY products mean that SeY is not a single nutritional entity [15]. For engineered forms, incomplete reporting of particle size, surface chemistry, and colloidal behavior remains a major blind spot [8, 11]. Because these properties can alter bioavailability, tissue distribution, and risk even at identical nominal doses, insufficient source definition continues to limit meaningful cross-study comparison.

A second limitation is the uneven alignment of study objectives, endpoint hierarchy, and reporting depth across studies. Recent poultry studies increasingly use challenge-defined models and more targeted outcome sets, which is an important advance [10, 12, 25, 136]. However, cross-study comparability remains limited because primary biological objectives are not always specified with equal clarity, basal Se background and source identity are not reported consistently enough for robust comparison, and antioxidant-related improvements are still sometimes overextended as broad surrogates of efficacy across distinct goals. Future studies should pre-specify one primary target outcome, report basal dietary Se background and source identity in a comparable manner, and organize secondary indices around that target rather than treating descriptive marker panels as interchangeable evidence of benefit.

Future progress should depend less on comparing ever more Se sources in parallel and more on testing defined source classes against defined biological questions. Priority should be given to studies that specify the intended production or physiological goal in advance, report exposure identity and basal Se background rigorously, use layered monitoring across exposure, status, and function, and evaluate whether apparent source advantages are reproducible across challenge conditions and production stages. Such a shift would move poultry selenium nutrition from broad source comparison toward truly goal-based precision design.

## Conclusions

This review emphasizes that poultry selenium nutrition should be interpreted through source identity, exposure definition, and goal-specific efficacy rather than total dietary selenium alone. Inorganic salts remain appropriate for baseline adequacy, whereas reserve-forming SeMet-type inputs are generally better supported for prolonged stress buffering and more predictable biofortification. Microbial and engineered systems may provide context-specific advantages, but their effects remain more product- and formulation-dependent. Importantly, maximal selenium deposition does not necessarily coincide with maximal functional benefit. Precision selenium use therefore requires matching source and dose to the intended production or physiological goal and evaluating outcomes with goal-relevant endpoints. Future progress will depend on better source characterization, clearer endpoint alignment, and more comparable reporting across studies.

## Supplementary Information

Additional file 1: Table S1. Comparative framework of selenium sources in poultry.

Additional file 2: Table S2. Key reporting elements to improve cross-study comparability of selenium interventions in poultry.

## Data Availability

No datasets were generated or analysed during the current study.

## Abbreviations

AFB_1_: Aflatoxin B_1_
Akt: Protein kinase B
ARE: Antioxidant response element
ATF6: Activating transcription factor 6
CAT: Catalase
CHOP: C/EBP homologous protein
DIO: Iodothyronine deiodinase
ER: Endoplasmic reticulum
GPx: Glutathione peroxidase(s)
GRP78: Glucose-regulated protein 78
IFN-γ: Interferon-gamma
IgA: Immunoglobulin A
IGF-1: Insulin-like growth factor 1
IgG: Immunoglobulin G
IgM: Immunoglobulin M
IL-1β: Interleukin-1 beta
IL-2: Interleukin-2
IL-6: Interleukin-6
Keap1: Kelch-like ECH-associated protein 1
MDA: Malondialdehyde
mTOR: Mechanistic target of rapamycin
Muc2: Mucin 2
MyD88: Myeloid differentiation primary response 88
NF-κB: Nuclear factor kappa B
NLRP3: NOD-like receptor family pyrin domain containing 3
Nrf2: Nuclear factor erythroid 2-related factor 2
OH-SeMet: Hydroxy-selenomethionine
PI3K: Phosphoinositide 3-kinase
pIgR: Polymeric immunoglobulin receptor
PTEN: Phosphatase and tensin homolog
ROS: Reactive oxygen species
SCLY: Selenocysteine lyase
Se: Selenium
Sec: Selenocysteine
SECIS: Selenocysteine insertion sequence
SeCys: Selenocysteine
SeMet: Selenomethionine
SeNPs: Selenium nanoparticles
SEPHS1: Selenophosphate synthetase 1
SeY: Selenium yeast
SHMT1: Serine hydroxymethyltransferase 1
sIgA: Secretory immunoglobulin A
SOD: Superoxide dismutase
SS: Sodium selenite
T-SOD: Total superoxide dismutase
TAC: Total antioxidant capacity
TBARS: Thiobarbituric acid reactive substances
Th1: T helper 1
TLR: Toll-like receptor
TRIF: TIR-domain-containing adapter-inducing interferon-β
TrxR: Thioredoxin reductase(s)
TXNRD1: Thioredoxin reductase 1
UPR: Unfolded protein response
WHC: Water-holding capacity
