# Supplementary material for: Form-specific selenium supplementation in poultry: linking bioefficacy to mechanisms and precision applications
Source: J Anim Sci Biotechnol. 2026 Jun 13;17:118. doi: 10.1186/s40104-026-01436-5 (PMC13263945; doi:10.1186/s40104-026-01436-5)
Supplement: Supplementary file 1 — Additional file 1: Table S1. Comparative framework of selenium sources in poultry. [file 40104_2026_1436_MOESM1_ESM.docx]

**Table S1. Comparative framework of selenium sources in poultry^1^**

| **Feature** | **Inorganic Se** | **SeMet-type organic Se** | **Biotransformation-derived Se^2^** | **Engineered nano-Se** | **Carrier-bound / functionalized Se** |
| --- | --- | --- | --- | --- | --- |
| **Representative forms** | Sodium selenite; sodium selenate | L-selenomethionine (SeMet); OH-SeMet; SeMet-rich products | Se-yeast; Se-enriched probiotics/microbes; Se-algae (mixed species) | Selenium nanoparticles (SeNPs), chemically or biologically synthesized; coated/modified | Chitosan–Se, polymer–Se, protein–Se conjugates; other carrier-complexed Se |
| **Intestinal uptake (simplified)** | Selenite: mainly passive diffusion; selenate: sulfate co-transporters | Amino-acid transporters (shared with methionine pathways) | Digestion releases SeMet/SeCys species → amino-acid transporters; matrix/carrier effects possible | Particle-associated uptake (e.g., endocytosis/macropinocytosis) and/or partial dissolution; strongly formulation-dependent | Controlled release and/or mucoadhesion-enhanced interaction; uptake depends on carrier design |
| **Metabolic fate & retention** | Rapid reduction/metabolism; typically lower retention; limited protein-bound reserve formation | Non-specific substitution for Met in body proteins → protein-bound reserve pool; generally higher and more sustained retention | Fate depends on speciation profile and matrix; retention variable across products/batches | Retention/distribution strongly formulation-specific; may show altered tissue partitioning vs molecular Se | Fate and retention governed by carrier chemistry and release kinetics; highly design-dependent |
| **Key advantages** | Cost-effective; reliable for meeting baseline adequacy; long history of use | Builds protein-bound reserves; more stable long-term status; predictable egg/meat biofortification | Co-delivers matrix bioactives; potential gut modulation and sustainability value (carrier + Se effects) | Potential mucosal-interface effects; may improve barrier/inflammation markers in some models (formulation-dependent) | Improved stability and controlled delivery; potential for precision targeting (design-dependent) |
| **Key limitations** | Limited reserves; lower deposition vs SeMet-type; narrower safety buffer under high oxidative load; pro-oxidant risk if mis-dosed | Higher cost; risk of excessive accumulation with prolonged high dose; product-to-product variability (esp. yeast-based) | High variability in speciation/matrix; requires rigorous characterization; comparability/regulatory challenges | Bioavailability and efficacy are endpoint-dependent; requires particle characterization; safety concerns at high dose/prolonged exposure | Synthesis complexity; standardization and regulatory pathway may be demanding; cost/scale-up constraints; requires thorough characterization |
| **Best-fit applications** | Deficiency correction; baseline supplementation (within regulations) | Long-term status stability; stress buffering (endpoint-dependent); egg/meat biofortification | Gut barrier/microbiota-oriented programs; products where matrix effects are desired and characterized | Challenge-specific gut health/inflammation programs; validated formulations; research/controlled application | Precision or research settings requiring controlled release/targeting; validated carrier designs |

^1^ Comparisons are context-dependent and may vary with speciation, dose, baseline Se status, diet composition, and (for engineered systems) formulation descriptors.

^2^ SeY treated as biotransformation-derived due to mixed speciation and matrix effects.
