# Supplementary material for: Form-specific selenium supplementation in poultry: linking bioefficacy to mechanisms and precision applications
Source: J Anim Sci Biotechnol. 2026 Jun 13;17:118. doi: 10.1186/s40104-026-01436-5 (PMC13263945; doi:10.1186/s40104-026-01436-5)
Supplement: Supplementary file 2 — Additional file 2: Table S2. Key reporting elements to improve cross-study comparability of selenium interventions in poultry. [file 40104_2026_1436_MOESM2_ESM.docx]

**Table S2. Minimum reporting elements to improve cross-study comparability of selenium interventions in poultry^1^**

| **Category** | **Examples** | **Report at minimum (for comparability)** |
| --- | --- | --- |
| Inorganic salts | Sodium selenite (SS); sodium selenate | Chemical form; purity/supplier; analyzed total Se in feed/water; background diet Se; major co-supplements (e.g., vitamin E, Zn, S). |
| SeMet-type organic Se | SeMet; L-SeMet; OH-SeMet | Chemical identity/isomer where relevant; purity/supplier/product specification; analyzed total Se in finished feed/water; batch/lot information if available; basal diet Se and major co-supplements. |
| Biotransformation-derived matrices | Se-yeast (SeY); other microbial biotransformation carriers (Se-enriched *Bacillus*/*Lactobacillus* products etc.) | Analyzed total Se; available speciation/QC (SeMet%, other Se-amino acids, Se⁰, residual oxyanions); strain/process/product specification; batch/lot information; strain identity; enrichment/culture conditions; whether live cells/spores/cell-wall fractions remain; relevant co-metabolites or carrier properties if invoked. |
| Plant-enriched Se | Se-enriched *Cardamine* spp. (SEC/SeCv) | Plant species/part; enrichment protocol and Se precursor; analyzed total Se; speciation profile (SeMet/MeSeCys/SeCys, as available); key co-phytochemicals if used for mechanistic claims. |
| Nano/engineered Se | Biogenic/green SeNPs; glycine-SeNP; coated/loaded SeNPs | Core chemistry/oxidation state; impurities/residual reagents; size distribution and morphology; surface chemistry/coating; zeta potential; stability in stock and finished feed/water; dissolution/release; batch-to-batch variability. |
| Functionalized/complexed Se | Selenized glucose; selenide chitosan; other Se–carrier conjugates | Chemical linkage/structure; carrier MW/degree of substitution; analyzed total Se; free vs bound fraction; stability and release under processing/digestion conditions. |
| Combination strategies | Se + butyrate/curcumin/Vit E; mixed SS+organic Se | Dose of each component and route; predefined primary endpoint; minimal monitoring set; avoid attributing effects to Se alone without reporting co-interventions. |

^1^ This table is a pragmatic comparability checklist (not a formal reporting guideline).
